# Nanomaterials-Based Optical Techniques for the Detection of Acetylcholinesterase and Pesticides

**DOI:** 10.3390/s150100499

**Published:** 2014-12-30

**Authors:** Ning Xia, Qinglong Wang, Lin Liu

**Affiliations:** 1 College of Chemistry and Chemical Engineering, Anyang Normal University, Anyang 455000, China; E-Mail: xianing82414@csu.edu.cn; 2 Henan University of Animal Husbandry and Economy, Zhengzhou 450046, China; E-Mail: yxyanghxx@gmail.com

**Keywords:** acetylcholinesterase, pesticides, nanomaterials, optical sensors

## Abstract

The large amount of pesticide residues in the environment is a threat to global health by inhibition of acetylcholinesterase (AChE). Biosensors for inhibition of AChE have been thus developed for the detection of pesticides. In line with the rapid development of nanotechnology, nanomaterials have attracted great attention and have been intensively studied in biological analysis due to their unique chemical, physical and size properties. The aim of this review is to provide insight into nanomaterial-based optical techniques for the determination of AChE and pesticides, including colorimetric and fluorescent assays and surface plasmon resonance.

## 1. Introduction

**Figure 1.**
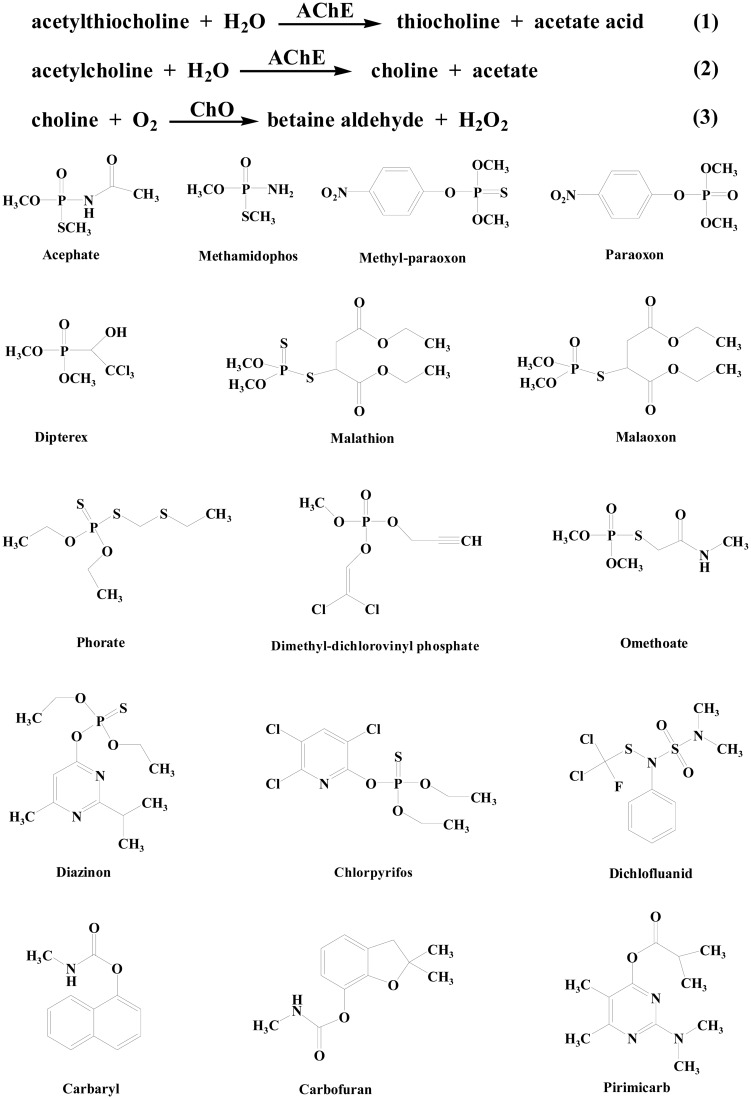
Structures of the main pesticides used as targets in AChE-based biosensors.

Acetylcholinesterase (AChE) catalyzes the hydrolysis of the neurotransmitter acetylcholine (ACh) to inactive choline. Inhibition of AChE will lead to the accumulation of acetylcholine in the synaptic cleft, resulting in impeded neurotransmission. AChE has regained great attention recently due to its association with Alzheimer's disease (AD) and other neurodegenerative diseases characterized by low ACh levels owing to catabolism by AChE [1,2]. Moreover, AChE is the primary target of inhibition by organophosphorus compounds such as nerve agents and pesticides (Figure 1); thus, public concern about the development of detection devices for effectively monitoring pesticides has also grown steadily [3–8].

The combination of enzymatic reactions with the various methods of monitoring enzymatic products has allowed the development of enzyme-based devices for sensitive and rapid determination of acetylcholine, AChE and its inhibitors [9–14]. In line with the rapid development of nanotechnology, nanomaterials have attracted great attention and have been intensively studied in biological analysis and detection due to their unique chemical, physical and size properties [15,16]. Commonly, nanomaterials can be employed for development of AChE-based sensing devices in the following three ways: (1) nanomaterials are used as enzyme carriers for loading a large amount of AChE to enhance the detection signal [17,18], especially in the electrochemical detection protocols, (2) nanomaterials act as peroxidase- or oxidase-like activities catalysts to catalyze the oxidation of various substrates including 2,2′-azino-bis(3-ethylbenzo-thiazoline-6-sulfonic acid) diammonium salt and 3,3,5,5-tetramethylbenzidine (TMB) by enzyme-generated hydrogen peroxide (H_2_O_2_) for colorimetric or fluorescence detection of acetylcholine and AChE inhibitors [19,20], and (3) nanomaterials are employed as the direct signal sources [21–23]. Electrochemical techniques based on the inhibition of AChE are attractive for the detection of acetylcholine and AChE inhibitors. In a typical configuration, the enzyme is deposited onto the surface of the working electrode. The activity of AChE is then monitored by adding the substrate to the solution and follow-up measuring the redox current of the enzymatic products. To improve the performance characteristics of the AChE-based electrochemical methods, electrode materials including nanomaterials have allowed for large quantities of enzyme to be immobilized, provide a favorable microenvironment to maintain the enzyme activity, and facilitate the oxidation of the enzymatic products. Nanomaterials used for the fabrication of AChE-based electrochemical biosensors have been summarized in recent review papers [10,24–26]. In this work, we highlighted the progress in development of nanomaterials-based optical techniques for the determination of AChE and pesticides.

## 2. Nanomaterials-Based Optical Techniques for Detection of Acetylcholine and AChE Inhibitors

The AChE-based sensing systems include the use of AChE alone or combination with choline oxidase (ChO). The AChE inhibition in the single and bienzyme systems is monitored by determining the generated enzyme production. AChE can hydrolyze acetylcholine or acetylthiocholine (a synthesized analogues of acetylcholine) to produce choline or thiocholine (a thiol compound), respectively. In the single enzyme system, AChE hydrolyzes acetylthiocholine to produce the thiocholine (Equation (1)). In the bienzyme system, AChE catalyzes the hydrolysis of acetylcholine into acetate and choline (Equation (2)). The choline is subsequently converted by ChO, producing hydrogen peroxide in the presence of oxygen (Equation (3)). Traditional optical methods to measure the levels of AChE and its inhibitors include the spectrophotometric thiol assay by using Ellman's reagent and the colorimetric detection of H_2_O_2_ produced by oxidation of the AChE-induced choline by using horseradish peroxidase (HRP) [27,28]. However, the methods lack sufficient sensitivity and require time-consuming sample-handling procedures. In order to enhance the detection sensitivity, advanced techniques based on metallic/magnetic nanoparticles and quantum dots have been developed recently, including colorimetric and fluorescent assays and surface plasmon resonance. Their preparation, modification and detection principle are presented herein.

### 2.1. Colorimetric Assays

**Figure 2.**
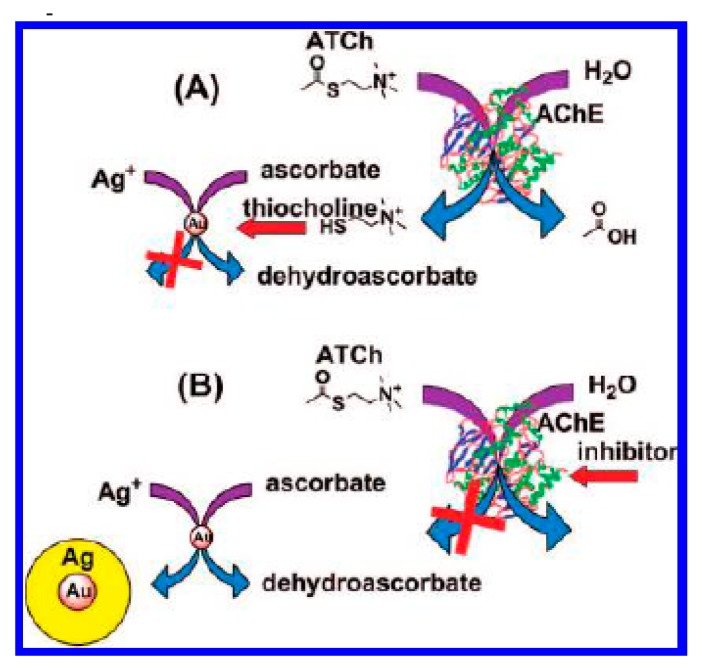
Detection of acetylcholine esterase inhibitors by growing silver-coated gold nanoparticles. Reprinted with permission from [34]. Copyright 2009 American Chemical Society.

Because of the high extinction coefficients and the unique size-dependent optical properties of gold nanoparticles (AuNPs), AuNPs-based colorimetric assays have recently become useful for many types of analytes without the need for advanced instruments, including screening the enzyme activity and measuring the concentrations of nucleic acid, proteins, metal ions and other small molecules [29–33]. In this process, molecular events can be transformed into color changes. Usually, the color changes are highly sensitive to the size, shape, capping agents, and medium refractive index, as well as the aggregation states of AuNPs, which can be confirmed by the significant absorption band shift in the visible region of the electromagnetic spectrum. Based on the unique physical properties of AuNPs, Pavlov *et al.*, presented the first colorimetric detection of AChE inhibitors based on the color change of AuNPs [21]. In the work, AChE mediated hydrolysis of acetylthiocholine to yield a reducing reagent thiocholine that modulated the growth of AuNPs seeds in the presence of AuCl_4_^−^. The catalytic growth of AuNPs was prevented by inhibition of AChE activity using 1,5-bis(4-allyldimethyl-ammoniumphenyl)-pentane-3-one dibromide or paraoxon, thus enabling a colorimetric assay for AChE inhibitors. However, this method is less sensitive because the enzymatic generation of AuNPs would consume thiocholine produced in the course of enzymatic reactions. Thus, the authors developed another method for the detection of AChE inhibitors based on the modulation of AuNPs growth by the enzymatically generated thiocholine [34]. As shown in Figure 2, the produced thiocholine hindered the deposition of Ag reduced by ascorbic acid from AgNO_3_ by binding to the surface of the Au seeds. As a result, the formation of Ag-coated AuNPs is blocked (route A). In the presence of AChE inhibitors, hydrolysis of acetylthiocholine by AChE was prevented, which allowed for the deposition of Ag on AuNPs surface (route B).

**Figure 3.**
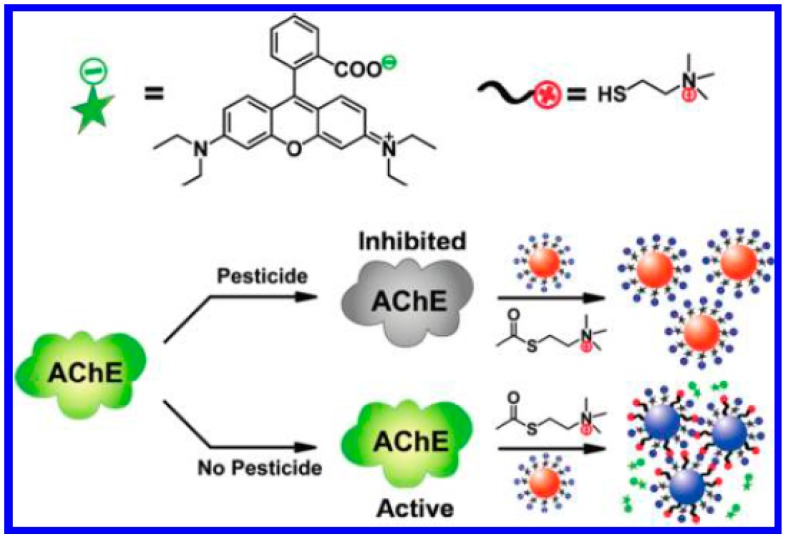
Design of the dual-readout (colorimetric and fluorometric) assay for pesticides. Reprinted with permission from [37]. Copyright 2012 American Chemical Society.

The modulation of aggregation states of AuNPs has also been broadly used as a colorimetric assay for various analytes. With the continual aggregation of AuNPs, the color of the dispersion gradually changes from an initial red to purple, then blue and, finally, yellow. The color change of AuNPs suspension can be confirmed by the significant absorption band shift in the visible region of the electromagnetic spectrum. For this view, Wang *et al.* demonstrated that the positively charged thiocholine can substitute the citrate on the surface of AuNPs; as a result, the cross-linking/aggregation of interparticles occurs because of the electrostatic interaction between thiocholine and citrate on gold surface, leading to the red-shift of the plasmon absorption of AuNPs suspension [35]. The degree of AuNPs aggregation is strictly dependent on the concentration of the produced thiocholine; thus, AChE activity could be monitored by the AuNPs-based colorimetric assay. With the method, AChE at the concentration as low as 0.6 mU/mL and tacrine (a well-known inhibitor for AChE) below 4 nM can be readily assayed. With the same principle, Sun *et al.*, reported the detection of organophosphate (OP) nerve agents and pesticide using lipoic acid (LA)-capped AuNPs with a pM detection limit [36]. Furthermore, Liu *et al.*, reported the assays of AChE in the cerebrospinal fluid of transgenic mice suffering from Alzheimer's disease and four pesticides (carbaryl, diazinon, malathion and phorate) using rhodamine B (RB)-functionalized AuNPs (RB-AuNPs) as the dual (colorimetric and fluorometric) readouts [37–39]. Specifically, electrostatic absorption of RB onto the surface of AuNPs leads to the quenching of RB's fluorescence (Figure 3). Thiocholine produced from acetylthiocholine by AChE substitutes RB on the surface of AuNPs, resulting in the color change of the solution from red to purple, simultaneously accompanied by the recovery of fluorescence of RB. The detection limit for AChE reaches 0.1 mU/mL and the lowest detectable concentrations for carbaryl, diazinon, malathion, and phorate are 0.1, 0.1, 0.3 and 1 μg/L, respectively.

Besides AuNPs, AgNPs have also been employed by Li *et al.*, for organophosphorus pesticide detection based on the thiocholine-induced aggregation of the citrate-stabilized AgNPs [40]. Similarly, organophosphorus pesticides prevented the production of thiocholine by inhibiting the activity of AChE, thus holding back the aggregation of AgNPs. As a result, a detection limit of 0.18 ng/mL for dipterex was achieved.

Fe_3_O_4_ magnetic nanoparticles (MNPs) exhibit peroxidase activity that can catalyze the oxidation of peroxidase substrates in the presence of H_2_O_2_ to produce a color reaction [41]. Liang *et al.* reported a Fe_3_O_4_ MNPs-based colorimetric method for the detection of organophosphorus pesticides and nerve agents using AChE and CHO [19]. In this method, AChE and CHO catalyzed the production of H_2_O_2_ in the presence of acetylcholine, which then activated MNPs to catalyze the oxidation of colorimetric substrate TMB to produce a color reaction. Inhibition of AChE by organophosphorus pesticides (acephate and methylparaoxon) and the nerve agent Sarin prevented the production of H_2_O_2_, resulting in a reduced catalytic oxidation of TMB and a decrease in the color intensity. Acephate, methylparaoxon and Sarin at the concentrations below 1 nM, 10 nM and 5 μM, respectively, can be readily detected.

### 2.2. Fluorescent Assays

#### 2.2.1. Quantum Dots

**Figure 4.**
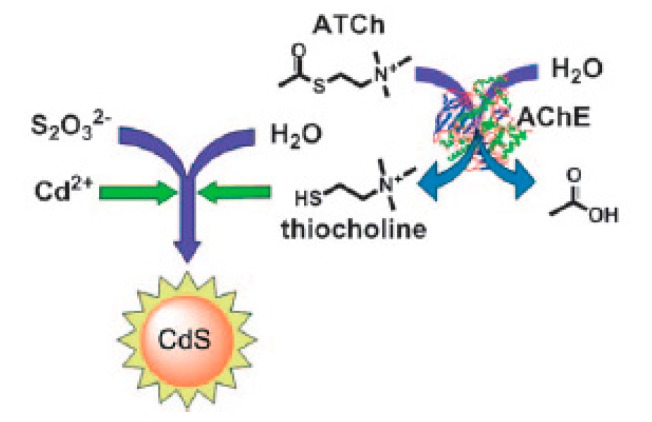
Enzymatic generation of CdS QDs for the detection of AChE activity. Reprinted with permission from [22]. Copyright 2010 John Wiley and Sons.

Comparing the chromatography evaluations and electrochemical analysis methods that need either time-consuming operation or complicated labeling and modification procedures, fluorimetric methodologies stand out as rapid, sensitive, and efficient especially in combination with the nanotechnologies and fluorescent nanomaterials. Semiconductor quantum dots (QDs) are the commonly known nanoparticles used in fluorescent sensing. The most important advantages of QDs over organic fluorophores are higher brightness, reduced photobleaching and longer lifetimes. Recently, several groups have reported the QDs-based fluorescence assays for detection of AChE activity and organophosphorus pesticides [22,42–46]. Typically, Pavlov's group demonstrated that thiocholine released from the AChE-catalyzed hydrolysis of acetylthiocholine (ATCh) are able to catalyze the production of fluorescent CdS QDs in the presence of thiosulfate and Cd^2+^ (Figure 4) [22] and can mediate stabilization of *in situ* produced CdS quantum dots [46]. As a result, AChE activity and its inhibitors can be determined by the fluorescence intensity of the resulting CdS QDs.

Silicon quantum dots (SiQDs), as inert, nontoxic, abundant, and low-cost nanomaterials, have been demonstrated to be environmentally friendly photoluminescence probes and have attracted much interest. In comparison to other QDs, SiQDs have unique optical and electronic properties, especially favorable biocompatibility. Yi *et al.* found that the fluorescence of label-free SiQDs could be effectively quenched by enzyme-generated H_2_O_2_ [47]. For this view, they further developed a SiQDs-based sensor for pesticides detection based on the fluorescence quenching of SiQDs induced by the enzyme-generated H_2_O_2_ [48]. Specifically, AChE hydrolyzed acetylcholine to choline; choline was then enzymatically oxidized by ChOx to produce betaine and H_2_O_2_. If the activity of AChE was inhibited by pesticides, the amount of the generated H_2_O_2_ would reduce, resulting in an increase in the fluorescence of SiQDs. The method allowed for the detection of carbaryl, parathion, diazinon and phorate at the concentrations below 7.25 ng/L, 32.5 ng/L, 67.6 ng/L and 0.19 mg/L, respectively. Additionally, Shen *et al.* found that the fluorescence of core-shell silica particles with tetraphenylethylene moieties could be quenched by dabcyl-ACh due to the electrostatic interaction between the silica particles and dabcyl-ACh [49]. After incubation with AChE, dabcyl-ACh was degraded between the residues of dabcyl and ACh, which caused the removal of dabcyl residues from the silica surface and the recovery of fluorescence of silica particles.

**Figure 5.**
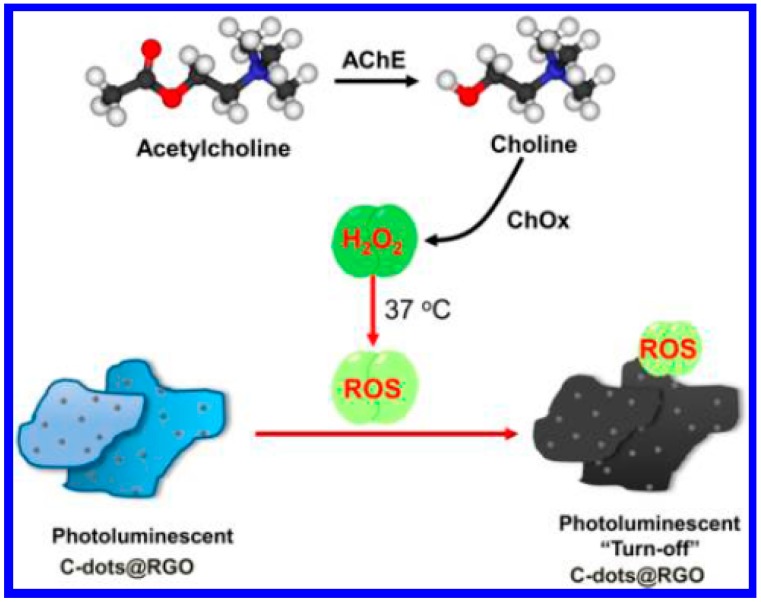
Schematic representation of sensing strategy of PL-quenched assay that uses C-dots@RGO for detection of ACh. Reprinted with permission from [51]. Copyright 2013 American Chemical Society.

Reduced graphene oxide (RGO) has become a very popular sensing material for the detection of DNA, proteins, and small molecules because of its large planar surface and high photoluminescence quenching efficiency to fluorophores (e.g., organic dyes, quantum dots) [50]. However, as-prepared RGO is usually hydrophobic and nonphotoluminescent, thus limiting its direct use for biological application [51]. Recently, Chang's group reported a strategy for the synthesis of hydrophilic, photoluminescent (PL) carbon dots on RGO (C-dots@RGO) from graphene oxide (GO) through a hydrothermal reduction route using catechin as a reductant [52,53]. Furthermore, they found that the AChE/ChOx-mediated production of H_2_O_2_ caused the photoluminescent quenching of the C-dots@RGO via an etching process (Figure 5) [51]. The photoluminescent intensity of the C-dots@RGO is inversely proportional to the acetylcholine concentration in the range of 0.05−10 nM, with a detection limit of 30 pM. By this method, the concentrations of acetylcholine in plasma and blood samples were determined to be 2.6 nM and 6.8 nM, respectively.

#### 2.2.2. Metal Nanoparticles

**Figure 6.**
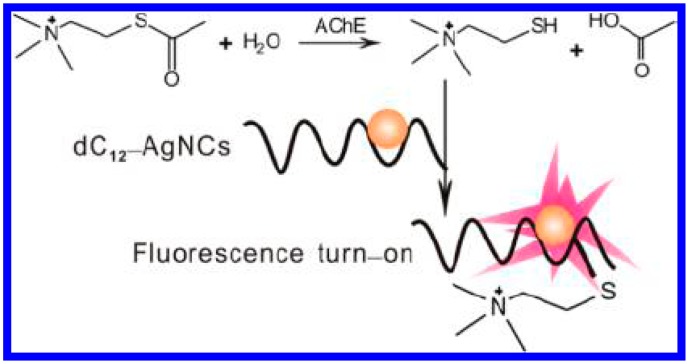
Principal reactions of fluorescence assay for AChE activity. Reprinted with permission from [55]. Copyright 2013 American Chemical Society.

The emerging technology of fluorescent few-atom metal (e.g., Au or Ag) nanoclusters (NCs) offers an attractive compromise between the photostability and brightness of quantum dots and the compact versatility of dye fluorophores [54]. This technology has recently been used in a wide range of chemical or biological detection and cellular imaging applications. For this consideration, Li *et al.* developed a fluorometric sensor for the detection of AChE activity and its inhibitors [23]. The method was based on the thiocholine-induced fluorescence quenching of DNA-templated copper/silver nanoclusters (DNA-Cu/AgNCs). The AChE activity could be detected as low as 0.05 mU/mL and with a linear range from 0.05 to 2.0 mU/mL. Inversely, Zhang *et al.*, found that the reaction of thiocholine to 12 polycytosine-templated silver nanoclusters (dC_12_−AgNCs) through the formation of Ag−S bonds lead to the increase of fluorescence of dC_12_−AgNCs (Figure 6) [55]. The hydrolysis of ATCh chloride was retarded in the presence of the corresponding inhibitor. Thus, AChE activity and its inhibitors could be determined with dC_12_−AgNCs as the fluorescence probes. This method allowed for the assay of AChE as low as 0.05 mU/mL. Moreover, based on the interaction of thiocholine and Ag(I), Liao *et al.* reported a “turn-on” fluorescent method for probing of AChE activity and sensing of AChE inhibitors. Specifically, a polyanion (poly(vinyl sulfonate)–PVS) could induce the aggregation and fluorescence quenching of a perylene probe (probe 1). The produced thiocholine interacted with Ag(I) to form a positively charged metal coordination polymer. As a result, the polycation interacted with the polyanion and caused the release of the free probe 1 monomer molecules, and a fluorescence turn on signal was detected [56].

Beside Ag NCs, Au NCs have also been extensively studied because of their intrinsic characteristics such as ease of preparation and chemical stability. Recently, Li *et al.* synthesized the denatured bovine serum albumin (dBSA)-protected AuNCs and demonstrated their applications in the fluorescent detection of AChE activity in human serum [57]. Specifically, the fluorescence of AuNCs was quenched by the produced thiocholine due to the combination of thiocholine with the dBSA-AuNCs. The method showed a linear range of 0.005–0.15 U/mL for AChE with a detection limit of 0.02 mU/mL. Also, Zhang *et al.* found that bovine serum albumin (BSA)-stabilized gold nanoclusters (BSA-AuNCs) can be used as the fluorimetric reaction substrate for probing the activity and phosphorylation of AChE and detecting dimethyl-dichlorovinyl phosphate (DDVP) [58]. They suggested that thiocholine released from the AChE-catalyzed hydrolysis of ATC caused the aggregation and fluorescence reduction of BSA-AuNCs. With this method, DDVP could be determined with a detection limit of 13.67 pM.

**Figure 7.**
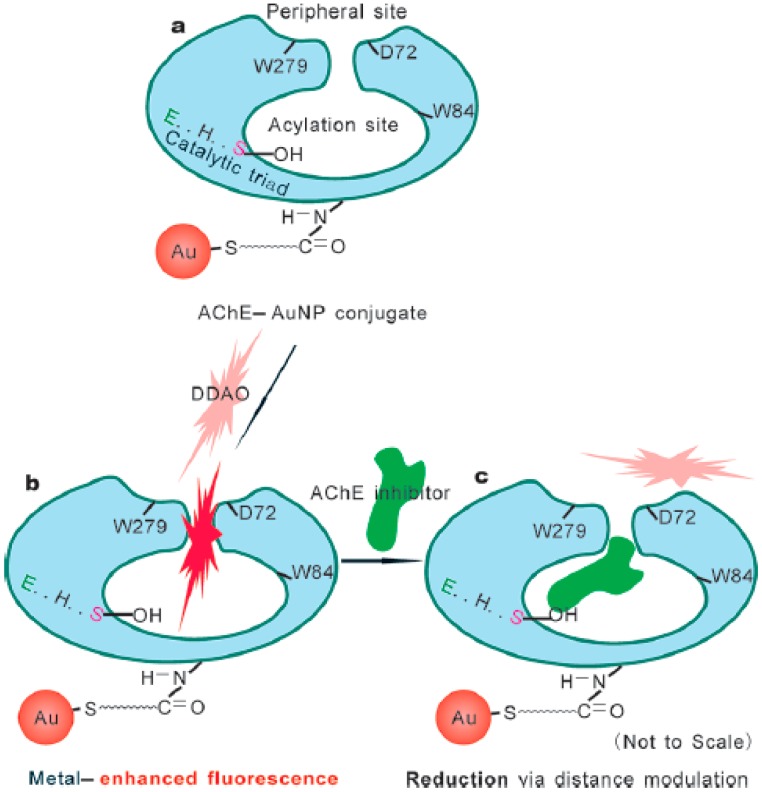
Schematic illustration of the strategy for the “on-off” type of fluorescent nanobiosensors for AChE inhibitors. (**a**) Fabrication of AChE-AuNP conjugate via EDC/NHS coupling chemistry; (**b**) Metal enhanced fluorescence of DDAO bound with the active sites of AChE via distance regulation between the fluorophore and AuNP; (**c**) The fluorescence reduction via distance modulation due to AChE inhibitor binding. Reprinted with permission from [63]. Copyright 2012 American Chemical Society.

Recently, there has been increasing attention paid to the use of metal NPs-based metal-enhanced fluorescence (MEF) for immunoassays, protein translation and DNA detection [59–62]. Enhancement is attributed primarily to the increased electric field close to the metal NPs induced by incident light. One of factors affecting the enhancement magnitude is the distance between fluorophores and metal nanostructures. Interestingly, Zhang *et al.* found that AChE could modulate the distance between AuNPs and fluorophore 7-hydroxy-9H-(1,3-dichloro-9,9-dimethylacridin-2-one) (DDAO) (Figure 7) [63]. Binding of DDAO to AChE immobilized onto AuNPs lead to the enhancement of DDAO's fluorescence due to MEF (Figure 7a). Because AChE inhibitors competed with DDAO to bind the peripheral anionic site and penetrate into the active gorge site of AChE, inhibition of AChE activity by AChE inhibitors such as paraoxon and tacrine prevented the interaction of AChE and DDAO, thus resulting in the distance variation between AuNPs and DDAO and reducing the fluorescence signal (Figure 7b). The results demonstrated that the biosensor shows low detection limits for paraoxon (0.4 μM) and tacrine (10 nM).

**Figure 8.**
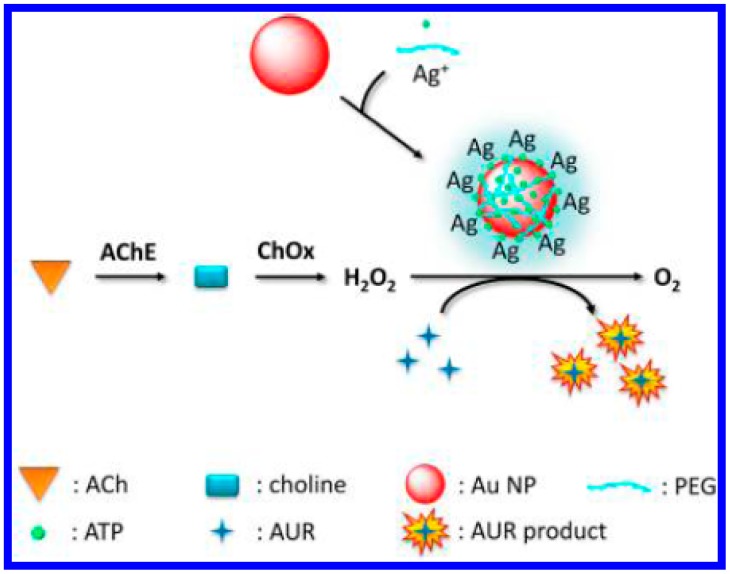
Schematic representation of the sensing strategy of the fluorescent assay using Au/Ag NPs to detect ACh. Reprinted with permission from [20]. Copyright 2012 American Chemical Society.

Moreover, nanoparticles (NPs) including Fe_3_O_4_, sheet-like FeS, spherical CeO_2_, single-walled carbon nanotubes, graphene oxide, AgM (M = Au, Pd, Pt) and metallic nanocomposites show peroxidase- or oxidase-like activities [20,41,64,65]. Chang's group suggested that Au/Ag bimetallic nanoparticles promoted the H_2_O_2_-mediated oxidation of Amplex UltraRed (AUR) and thus reported the fluorescent detection of acetylcholine (Figure 8) [20]. In this process, AChE catalyzes the hydrolysis of acetylcholine into acetate and choline. The choline is subsequently converted by ChO, producing H_2_O_2_ in the presence of oxygen. The as-produced H_2_O_2_ was made to react with AUR in the presence of Au/Ag bimetallic NPs. The detection limit of this method for acetylcholine was 0.21 nM.

### 2.3. Surface Plasmon Resonance

**Figure 9.**
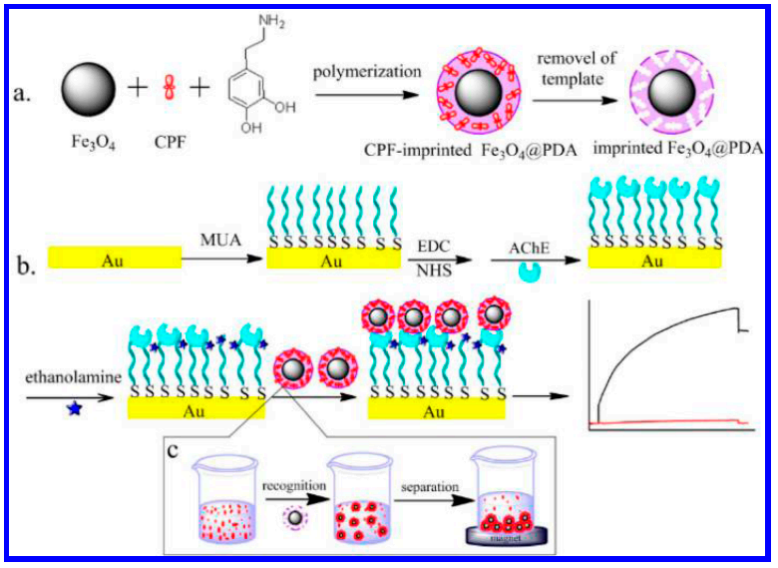
Schematic illustration of the strategy of ATP detection using AuNPs as indicators and Cu^2+^ ions as cross-linkers. Reprinted with permission from [70]. Copyright 2013 American Chemical Society.

SPR is the collective oscillation of electrons in a solid or liquid stimulated by incident light [66]. The resonance condition is established when the frequency of light photons matches the natural frequency of surface electrons oscillating against the restoring force of positive nuclei. SPR instruments optically monitor changes that occur on certain metal sensor surfaces (typically gold and silver) when sample fluid flows past the surface. Several SPR optical biosensors have been developed for detecting low levels of AChE inhibitors including pesticides residues [67–69]. However, because of the small size of the inhibitors, binding of the targets to the AChE-covered sensing chip dose not bring significant shift in the resonance angle. Thus, the use of the simple SPR method for pesticides detection shows a poor sensitivity. NPs can promote a significant shift in the angle of plasmon resonance. Qiu's group reported the synthesis of magnetic molecular imprinting polymers (MIPs) NPs with high density and accessible recognition sites for chlorpyrifos (CPF) [70]. The magnetic MIPs NPs were synthesized by self-polymerization of dopamine on the surface of Fe_3_O_4_ NPs in the presence of template CPF in weak base aqueous solution (Figure 9a). As a result, the target CPF molecules can be rapidly enriched and separated by the imprinted Fe_3_O_4_@polydopamine nanoparticles (Fe_3_O_4_@PDA NPs) by an external magnetic field. Integrating the CPF-imprinted Fe_3_O_4_@PDA NPs to a SPR chip through the specific interactions between the CPF rebound in the recognition cavities in the PDA matrix and the AChE immobilized on sensor chip results in a significant signal amplification due to the high molecular weight of Fe_3_O_4_@PDA NPs (Figure 9b). The SPR biosensor showed a low detection limit (0.76 nM) for CPF detection.

## 3. Conclusions/Outlook

In conclusion, we have reviewed the progress in the optical detection of AChE and pesticides using functional nanoscaffolds made of novel nanomaterials, such as metal and metal oxide nanoparticles, QDs and magnetic beads. Although most of the existing limitations of the AChE-based sensors could be directly related to the selectivity in multicomposite mixtures and complex matrices and the inability of identifying a specific pesticide, the sensitivity of most of the methods is sufficient to detect the minimum levels of total pesticides imposed by regulatory agencies. Moreover, the advances in nanoscience and nanotechnology promise a better future for designing of AChE-based biosensors that would complement or serve as an alternative to the existing expensive and complex chromatographic devices.
